# Extracellular vesicles coordinate mitochondrial trafficking between Leydig cells and testicular macrophages to sustain testosterone production

**DOI:** 10.20517/evcna.2026.61

**Published:** 2026-08-19

**Authors:** Marco G. Alves, Ruben J. Moreira, Ana Gabriela Henriques, Rita Ferreira, Pedro F. Oliveira

**Affiliations:** ^1^Institute of Biomedicine (iBiMED), Department of Medical Sciences, University of Aveiro, Aveiro 3010-193, Portugal.; ^2^LAQV-REQUIMTE, Department of Chemistry, University of Aveiro, Aveiro 3010-193, Portugal.

## MAIN TEXT

Testosterone serves as the primary endocrine determinant of male physiology, controlling several functions ranging from embryonic sexual dimorphism to the maintenance of adult musculoskeletal integrity and spermatogenesis^[1]^. In mammals, its synthesis predominantly occurs in Leydig cells (LCs), where testosterone production is coupled to high bioenergetic demand. Beyond fulfilling this bioenergetic requirement, the mitochondrion also hosts the initial and rate-limiting phase of steroidogenesis. This is defined by the StAR (steroidogenic acute regulatory) protein-mediated translocation of cholesterol across the intermembrane space to the inner mitochondrial membrane (IMM), where it undergoes enzymatic conversion into pregnenolone^[2]^. However, this metabolic specialization creates a persistent challenge. The high-flux steroidogenic signaling required for testosterone biosynthesis generates high levels of reactive oxygen species (ROS) as byproducts of both mitochondrial oxidative phosphorylation and cytochrome P450 enzymatic activity. Given that adult LCs are long-lived and exhibit low cellular turnover, they are very susceptible to cumulative oxidative damage affecting mitochondrial DNA (mtDNA), proteins, and lipid membranes^[3,4]^. Cell-intrinsic quality control mechanisms such as PTEN-induced kinase 1 (PINK-1)-mediated mitophagy are well-characterized^[5]^, but they may be insufficient to maintain continuous mitochondrial proteostasis and organelle integrity over time under sustained steroidogenic stress. Addressing this challenge, a recent study by Xia *et al*. describes a mechanism of transcellular homeostasis in the mouse testis, in which LCs maintain mitochondrial homeostasis by externalizing damaged mitochondria through structures termed extracellular vesicles (EVs)^[6]^. This finding must be contextualized within the rapidly expanding literature regarding intercellular mitochondrial transfer, a phenomenon increasingly recognized as a potential contributor to tissue homeostasis. Previous research elucidated different mechanisms for organelle exchange, including tunneling nanotubes, macrophage-mediated organelle clearance, mesenchymal stem cell (MSC) mitochondrial donation, and neuron-glia mitochondrial trafficking^[7,8]^.

Furthermore, the identification of extracellular mitochondria, involved in cell-cell communication, has added significant complexity to the understanding of intercellular communication. The study by Xia *et al*. extends these concepts to the testicular microenvironment, proposing a bidirectional mitochondrial transfer network wherein specific macrophage subpopulations coordinate both the clearance of defective LC mitochondria and the provision of functional replacements^[6]^. The initial stage of this mechanism involves the selective extrusion of compromised mitochondria via LC-derived particles, designated as LC-EVs. Although proteomic analysis of LC-derived particles identified a high percentage of proteins already catalogued in Vesiclepedia, no evaluation of EV parameters in accordance with the Minimal Information for Studies of Extracellular Vesicles (MISEV) criteria was performed on these particles. As highlighted by the MISEV guidelines, distinguishing such large EVs from apoptotic bodies, cellular debris, or entirely distinct extracellular structures is important for establishing the biogenesis pathways of these structures^[9]^. Future studies should therefore employ isolation protocols and complementary characterization approaches to minimize the co-isolation of non-vesicular material and establish the identity and purity of these extracellular particles, in accordance with the MISEV guidelines. Crucially, however, in the paper, the mitochondrial cargo and morphology display features consistent with mitochondrial dysfunction, including increased oxidative stress, reduced cristae area, dissipated membrane potential, and reduced ATP levels. Nonetheless, no bioenergetic analysis was performed to directly evaluate the functionality of LC-EV mitochondria or their functional impact on recipient cells. The authors reveal that the selective cargo sorting within LCs is governed by antagonistic motor proteins. The anterograde kinesin family member 5B (KIF5B) drives the displacement of damaged mitochondria toward the cortical periphery for export, whereas the myosin MYO6 acts as an anchor, retaining functional organelles within the cell soma. Under conditions of high oxidative stress inherent to steroidogenesis, defective mitochondria lose their MYO6 association, thereby favoring KIF5B-mediated trafficking into EVs. According to this work, following their release, LC-derived EVs are subsequently sequestered by a specific subpopulation of resident testicular macrophages, CD206^hi^ tMacs. This transcellular clearance is dependent on the triggering receptor expressed on myeloid cells 2 (TREM2) receptor, which likely recognizes externalized phosphatidylserine (PS) on the EV surface as a canonical clearance signal, a marker often upregulated in response to oxidative stress. The authors demonstrated that genetic ablation of TREM2 leads to an extracellular accumulation of mitochondrial debris. This accumulation is accompanied by impaired intracellular mitochondrial turnover within the LCs, compromising their proteostatic flux and ultimately resulting in impaired steroidogenesis. These findings support a role for this macrophage-dependent clearance axis in maintaining LC mitochondrial homeostasis and testosterone production.

A key finding of the Xia *et al*. study is that this cellular crosstalk operates as a bidirectional exchange rather than a simple unidirectional clearance pathway^[6]^. The authors demonstrate that LCs acquire mitochondria with preserved structural and functional features originating from a different, transcriptionally separated tMac subpopulation. Utilizing lineage-tracing models with fluorescently labeled macrophage mitochondria, they mapped the directional flux of these organelles into the LC cytoplasm. Unlike the degraded cargo observed in LC-derived EVs, the mitochondria sequestered from the tMACs expressing high levels of major histocompatibility complex class II (MHCII^hi^) tMacs exhibited a preserved ultrastructure with dense cristae. This intercellular organelle transfer is facilitated by a specific molecular bridging complex. The researchers identified that MHCII^hi^ tMac-secreted EVs are enriched with the integrin beta 1 (ITGβ1). This integrin selectively binds to the vascular cell adhesion molecule 1 (VCAM1) receptor expressed on the LC plasma membrane, facilitating docking and subsequent endocytosis of tMac-secreted vesicles [Figure 1]. Following internalization, these exogenous mitochondria appear to integrate into the LC network, leading to measurable increases in cellular ATP levels necessary to meet the demanding metabolic requirements of testosterone synthesis. Pharmacological or genetic antagonism of the VCAM1 axis precipitates a failure in this mitochondrial replenishment, compromising testosterone production. These findings support a functional contribution of transferred mitochondria, but they warrant a deeper methodological critique. The reliance on static, fluorescence-based ATP determination, rather than direct metabolic flux analyses or high-resolution respirometry, leaves the extent of functional mitochondrial integration partially unaddressed. An increase in intracellular ATP does not prove full structural integration into the host's oxidative phosphorylation network. Furthermore, critical questions regarding exogenous mtDNA persistence, cellular heteroplasmy dynamics, and evasion of recipient mitophagy remain unexplored. Thus, long-term functional validation will be essential to substantiate this model.

**Figure 1:**
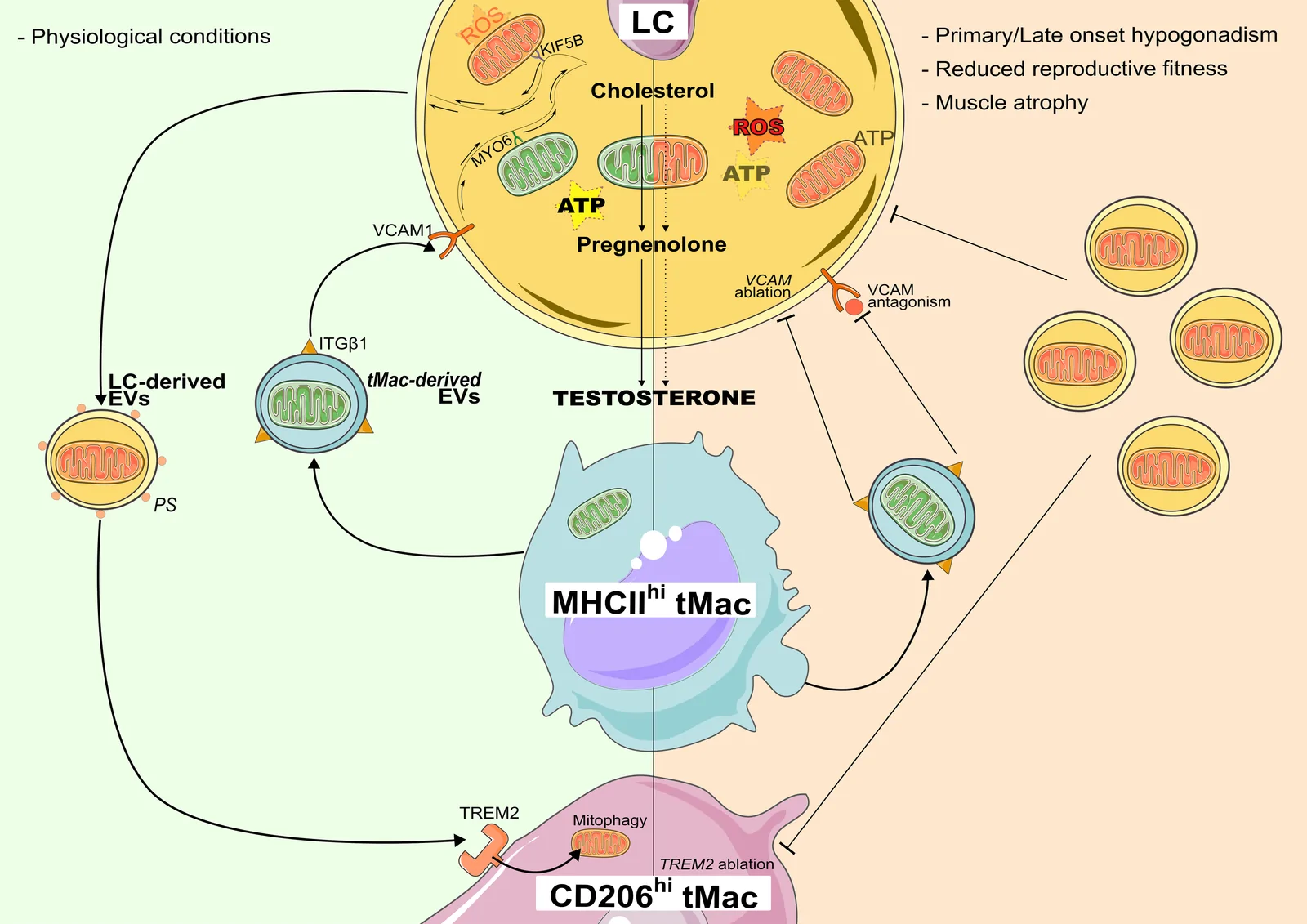
Proposed EV-mediated mitochondrial exchange between LC and tMacs. To manage the metabolic demands and ROS generated during steroidogenesis, LCs release extracellular particles containing damaged or functionally compromised mitochondria, which are taken up by CD206^hi^ tMacs. This clearance is governed by the TREM2 receptor recognizing surface-exposed PS. Selective cargo sorting utilizes KIF5B for export, while MYO6 retains healthy organelles. Concurrently, LCs internalize functional mitochondria from MHCII^hi^ tMacs via an ITGβ1-VCAM1 interaction. Disruption of this network leads to the accumulation of dysfunctional mitochondria, impairing testosterone production and mirroring features of hypogonadism and reduced reproductive fitness. Figure was created using stock images provided by *Servier Medical Art* (https://smart.servier.com), licensed under CC BY 4.0 (https://creativecommons.org/licenses/by/4.0/). ATP: Adenosine triphosphate; CD206: cluster of differentiation 206; EV: extracellular vesicle; ITGB1: integrin beta 1; KIF5B: kinesin family member 5B; LC: Leydig cell; MHCII: major histocompatibility complex class II; MYO6: myosin VI; PS: phosphatidylserine; ROS: reactive oxygen species; tMac: testicular macrophage; TREM2: triggering receptor expressed on myeloid cells 2; VCAM1: vascular cell adhesion molecule 1.

Despite these limitations, the study provides evidence that disruption of this transfer network has physiologically relevant consequences in mice. Targeted macrophage depletion, or the genetic ablation of either the *Trem2* or *Vcam1* signaling nodes, induced systemic endocrine and metabolic disruption in murine models. These mice exhibited a primary hypogonadism-like phenotype, evidenced by a significant decline in circulating testosterone levels; impaired reproductive fitness, characterized by reduced spermatogenesis and diminished sperm motility; and a musculoskeletal phenotype marked by *gastrocnemius* atrophy and a reduced endurance capacity. These phenotypes share clinical similarities with features of late-onset hypogonadism (LOH)^[10]^ observed in aging human populations^[11]^. In aged murine models, transcriptomic shifts downregulate TREM2, impairing clearance, while reduced VCAM1 limits bioenergetic replenishment. This dual failure, coupling the intracellular accumulation of pro-oxidant debris with reduced mitochondrial replenishment and ATP-generating capacity, may contribute to age-related steroidogenic decline. In summary, the study by Xia *et al*. supports a broader view of the testicular microenvironment, moving away from a strictly LC-centric perspective toward a co-dependent multicellular ecosystem^[6]^. By delineating the functional divergence among macrophage subpopulations, where CD206^hi^ and MHCII^hi^ lineages execute distinct roles in clearance and mitochondrial donation, respectively, the research provides a novel view of tissue homeostasis. Nevertheless, mouse models are limited for studying human steroidogenesis because of species-specific differences in LC regulation, steroid output, and mitochondrial dynamics, which may alter processes such as mitochondrial trafficking or extrusion. Additionally, EV-mediated intercellular mitochondrial transfer remains mechanistically complex and requires further standardization of EV isolation and molecular validation. Ultimately, this study provides new insights into EV-mediated organelle transfer in endocrine tissues while highlighting the need for further investigation into the molecular identity, biogenesis, and physiological significance of these mitochondria-containing extracellular particles.

## DECLARATIONS

### AI and AI-assisted tools statement

During the preparation of this manuscript, the AI tool Gemini (version 2.0 Flash, released 2025-02-05) was used solely for polishing the English language of this manuscript. The tool did not influence the study design, data collection, analysis, interpretation, or the scientific content of the work. All authors take full responsibility for the accuracy, integrity, and final content of the manuscript.

### Copyright

© The Author(s) 2026.
